# Decreased microRNA-132 and its function in human non-small cell lung cancer

**DOI:** 10.3892/mmr.2021.12161

**Published:** 2021-05-20

**Authors:** Xiyu Liu, Song Yan, Changyan Pei, Youbin Cui

Mol Med Rep 11: 3601-3608, 2015; DOI: 10.3892/mmr.2015.3222

Following the publication of this paper, it was drawn to the Editors’ attention by a concerned reader that the data shown in Fig. 5 and 6 were the same as those featured in another paper by different authors. Owing to the fact that the contentious data in the above article were already under consideration for publication elsewhere prior to its submission to *Molecular Medicine Reports*, the Editor has decided that this paper should be retracted from the Journal. After having been in contact with the authors, they agreed with the decision to retract the paper. The Editor apologizes to the readership for any inconvenience caused.

